# Preceding child survival status and its effect on infant and child mortality in India: An evidence from National Family Health Survey 2015–16

**DOI:** 10.1186/s12889-021-11569-z

**Published:** 2021-08-21

**Authors:** Shobhit Srivastava, Shubhranshu Kumar Upadhyay, Shekhar Chauhan, Manoj Alagarajan

**Affiliations:** 1 Department of Mathematical Demography and Statistics, International Institute for Population Sciences, Mumbai, India; 2 Senior Resource Person, Knowledge Resource management, IED/ UNICEF, Lucknow, India; 3 Department of Population Policies and Programmes, International Institute for Population Sciences, Mumbai, India; 4 Department of Development Studies, International Institute for Population Sciences, Mumbai, India

**Keywords:** Maternal and child health, Mortality

## Abstract

**Background:**

India has achieved impressive gains in child survival over the last two decades; however, it was not successful in attaining MDG 2015 goals. The study’s objective is to inquire how the survival status of the preceding child affects the survival of the next born child.

**Methods:**

This is a retrospective analysis of data from the National Family Health Survey, 2015–16. Analysis was restricted to women with second or higher-order births because women with first-order births do not have a preceding child. Proportional hazards regression, also called the Cox regression model, has been used to carry out the analysis. Kaplan–Meier (K–M) survival curves were also generated, with a focus on preceding birth intervals.

**Results:**

Results found that female children were more likely to experience infant mortality than their male counterparts. Children born after birth intervals of 36+ months were least likely to experience infant mortality. Mother’s education and household wealth are two strong predictors of child survival, while the place of residence and caste did not show any effect in the Cox proportional model. Infant and child deaths are highly clustered among those mothers whose earlier child is dead.

**Conclusion:**

Maternal childbearing age is still low in India, and it poses a high risk of infant and child death. Education is a way out, and there is a need to focus on girl’s education. The government shall also focus on raising awareness of the importance of spacing between two successive births. There is also a need to create a better health infrastructure catering to the needs of rich and poor people alike.

## Background

According to 2017 data, 4.1 million infants died, accounting for almost 75% of all fatalities among children under the age of five [1]. From 65 deaths per 1000 live births in 1990 to 29 deaths per 1000 live births in 2017, the global infant mortality rate has decreased [1]. Annual infant deaths have declined from 8.8 million in 1990 to 4.1 million in 2017 [1]. On the other hand, 5.4 million children under the age of five die in the same year, equating to 15,000 fatalities each day [2]. The risk of a child dying before completing five years of age is still highest in the WHO African Region (74 per 1000 live births), around eight times higher than that in the WHO European Region (9 per 1000 live births) [2]. Premature birth, pneumonia, congenital impairments, neonatal infections, malaria, sepsis, measles, delivery difficulties, and diarrhoea are all well-documented avoidable causes of mortality in children under five [3, 4].

Over the previous two decades, India has made significant advances in child survival; nonetheless, it failed to meet the MDG 2015 targets. In India, the U5MR has decreased by 3% each year since 1990, from 114 per 1000 live births in 1990 to 39 in 2016 [5]. Similarly, IMR decreased from 81 to 34 per 1000 live births between 1990 and 2016 [5]. Children born to mothers who had completed eight years of schooling had a better probability of surviving, but children born to adolescent mothers had a greater risk of dying [6]. Some preventative strategies for child health included exclusive breastfeeding, high-quality complementary foods, correct micronutrient feeding, vitamin A and iron, sufficient handwashing, and complete vaccination [7–9].

The previous study found that the effect of short preceding intervals on children under age five years was mainly concentrated in the neonatal period [10, 11]. Furthermore, previous research has shown that prenatal variables are more significant than postnatal ones in the causal pathway between early childhood mortality and early childbearing. If the gap between births was less than 24 months, the combined effects of pregnancy and breastfeeding on a mother’s physiology and nutritional condition might represent a mechanism through which short intervals impact child health. Low birth weight, preterm, and poor breastfeeding ability are all possible outcomes.

Even after adjusting for neonatal mortality, infants delivered after short birth intervals had a slightly greater probability of not being breastfed, which might imply that mothers who do not breastfeed have shorter birth intervals [12]. Longer birth intervals are associated with a decreased risk of child mortality. When the correlations were stratified by maternal completed fertility, the link between short birth intervals and child mortality vanished almost entirely for women with low completed fertility. Both shorter intervals and a higher risk of repeat infant mortality occur in families that have experienced one early infant death; the death of a second child after a short interval may not be due to the interval but to some other factor that also caused the first death and the shortened interval [13].

Short birth intervals may not deplete the mother’s nutritional resources to the degree that raises the child’s risk of death since low fertility women may have superior nutritional status and access to treatment. For high-fertility moms, nutritional status and access to treatment may be impacted from the outset [14, 15]. Multiple births are associated with a greater risk of mortality, according to numerous research. Furthermore, household wealth is a major predictor of infant mortality, but the mother’s education and age at first birth were not shown to be significant predictors of death [16].

Previous literature confirms that first-born children of adolescent mothers are the most vulnerable to infant mortality and poor child health outcomes [17]. This issue can be confirmed by the fact that low maternal age is highly associated with the stunting of the child [17]. Offspring mortality had a U-shaped association with maternal age, as compared to the reference group of 20–24 years, younger (≤19 years) and older (≥35 years) maternal ages were associated with a higher risk of offspring mortality [18]. However, the higher age of the mother contributes to an increased risk of preterm delivery, which poses a high risk of infant mortality to the male gender than their female counterparts [19]. Various prior research has shown that birth order and infant and child mortality risk have a substantial association. The birth-order impact is said to be significantly greater in the post-neonatal period. The chance of dying increases as the birth order rises, and this relationship is further divided between male and female children.

Furthermore, the mother’s age and education and the child’s sex and birth weight all play a role in understanding the link between birth order and infant mortality [20]. Early neonatal, neonatal, post-neonatal, and infant mortality are all likely to have a U–shaped association with the mother’s age at birth [21]. Even after accounting for the previous birth–to–conception gap, the child’s birth order, and other possible confounders, the U-shaped connection remains [21]. Women’s education contributes to child survival through increasing health knowledge and giving them more influence over their children’s health decisions, as well as preventing and controlling childhood illnesses [6, 8, 22–25]. Education attainment has been noted to improve social and economic standards, further determining maternal health care utilization [24].

India can play a key role in global efforts to stop the preventable death of newborns and children under the age of five since it has the greatest number of deaths linked to newborns and children under the age of five. In the study of newborn and child mortality, the previous child survival status is a significant predictor [25, 26], which further is linked to improved infant and child mortality [22]. Furthermore, previous studies have noted a higher prevalence of child death in families with a prior history of experiencing child loss [27, 28]. While there is a plethora of studies on the factors that influence newborn and child mortality, there is little data on the relationship between previous child survival status and infant and child death. Only a small amount of study has been done on the link between the death of the previous child and the following impact on infant and child mortality.

Zenger (1993) describes the mechanism that stems from the death of a child may plausibly raise the risk of death of his or her subsequent sibling [29]. She only estimated either the previous sibling’s survival status or mother-related unobserved heterogeneity, and not both [29]. However, some other studies have included both [30–33]. Bolstad & Manda (2001), utilizing Malawi Demographic and Health Survey, learned that infant and child deaths tend to cluster in some families with a history of a previous child [30]. However, Sastry (1997) uncovered that childhood mortality clustering is mainly attributed to birth spacing and that death of the previous child followed by a shorter birth spacing period would be a great risk for the subsequent child when investigating the pathways of family-level clustering of childhood mortality in Northeast Brazil [33].. Guo (1993), using sibling data to estimate family mortality effects in Guatemala, estimated that each child loss in the family was associated with a 22% increase in the risk of death to the index child [32]. The study from Guo (1993) was different from this study as that study could link the risk of death of index child to several previous birth histories whereas, in this study, the risk is linked to only previous birth history [32]. Curtis et al. (1993) noted that birth interval between two successive births was an important predictor of childhood mortality in Brazil [31]. The risk of death of an index child attributed to the death of a previous child was higher when the birth spacing was low [31].

In light of the foregoing explanation, this study aimed to see how the survival status of the previous child influences the survival status of the next born child. The present study hypothesized that there was no significant link between the survival status of the previous child and the survival status of the following born child.

## Methods

### Data

This is a retrospective data from the 2015–16 National Family Health Survey. The fourth in the NFHS series, the 2015–16 National Family Health Survey (NFHS-4), offers data on India’s population, health, and nutrition for each state and union territory [34]. All four NFHS surveys have been conducted under the stewardship of the Ministry of Health and Family Welfare (MoHFW), Government of India [34]. MoHFW designated the International Institute for Population Sciences (IIPS), Mumbai, as the nodal agency for all surveys [34]. The National Family Health Survey (NFHS) is a cross-sectional household survey performed on representative samples selected throughout India’s 36 states [34]. The birth history data set contains data on 1,315,617 infants born between 1970 and 2016 who were born to 476,619 women. On the entire birth history of mothers, we conducted two different analyses. The first analysis looked at newborn death, whereas the second looked at child death separately. In the previous five years, only singleton births were included in the study. Only children with birth orders of two or above are included in the sample. As a result, the study’s analytical sample size was 824,693 children aged 0 to 59 months in India.

### Variable description

#### Outcome variables

The current study had two outcome variables: infant (0–11 months) and child (12–59 months) mortality. Infant mortality refers to a child’s death before his first birthday, whereas child mortality refers to a child’s death from his first birthday to the day before his fifth birthday.

#### Explanatory variables

The key explanatory variables were any preceding child’s survival status (alive, dead), the child’s sex (male, female), maternal age at childbirth (18, 18–34, 35+), birth order (2, 3, and 4+), and the preceding birth interval (PBI), defined as the difference in months between the index child’s birth date and the preceding child’s birth date. It was categorized as a four-category variable (< 19 months, 18–23 months, 24–35 months, and 36 months), and the status of having multiple births was classified as (single and multiple). Other mothers socio-economic variables were taken into account, education (including illiteracy, primary, secondary, and higher education), religion (Hindu, Muslims, and Others), Caste (deprived: Scheduled Caste/Scheduled Tribe (SC/ST) and not-deprived: other than SC/ST), wealth index (poorest, poorer, middle, richer and richest), type of residence (urban and rural), regions of India (north, central, east, north-east, west and south).

### Statistical analysis

The unadjusted estimates were derived using bivariate analysis. To achieve the objectives of the study, Cox regression (also known as proportional hazards regression) was employed.

In this study, infant and child fatalities were utilised to conduct the analysis. The Kaplan–Meier (K–M) survival curves for the PBI were also calculated [16]. The dataset was analysed, and the model’s fitness was assessed using non-parametric K–M survival curves [16]. The index child’s age (in months) was used as the time variable, and the child’s death was used as the censoring variable in the Cox models [16]. Fotso et al. (2016) also defined the hazard of mortality at any point in time t is given as [16]:

$$ h\left(t|X\right)=h(t)\mathit{\exp}\sum \limits_{i=1}^p{X}_i{\beta}_i $$

Where h (t) is the baseline hazard, representing the probability of the child dying before any exposure to X [16].

In this study, the proportionality assumptions of all Cox models were evaluated. The Schoenfeld proportionality test was used in this study [16], a technique for re-estimating the Cox proportional hazard model using residuals [16]. The multivariate analyses were carried out through four models [16]. The major explanatory variables (survival status of any previous child) was represented as a binary variable (alive or dead) in Model 1. Model 2 incorporates model 1 as well as five additional bio-demographic variables: the child’s gender, maternal age at childbirth, PBI, and single/multiple births. Model 3 incorporates the mother’s remaining background variables, such as education, religion, caste, wealth index, type of residence, and regions, into model 1. All of the additional variables from all three models are combined in Model 4. The data reported in the study were analysed using STATA 13.

## Results

Table 1 shows the percentage of infant and child deaths by their background characteristics in India. Births succeeded by a dead child had the highest infant (15.21) and child deaths (2.86). Infant deaths constitute more deaths of male children (4.98), while child mortality was higher among females (1.61). Higher infant (5.61) and child deaths (1.63) were noticed for maternal age below 18 years. Noticeably, 4+ birth order was associated with higher infant (6.43) and child mortality rates (1.93). Births with preceding birth intervals shorter than 19 months had the highest infant (10.22) and child mortality rate (2.36). A considerably higher number of infants (26.61) and child deaths (1.82) were recorded in the case of multiple births. Among maternal socioeconomic factors, the education of mother and household wealth index showed a negative association with infant and child mortality rates. A significantly higher number of infant and child deaths were found among children belonging to the Hindu religion (5.04 & 1.39 respectively) and deprived section of the society (5.31 & 1.67 respectively). Higher infant (5.29) and child deaths (1.51) were found in the rural areas compared to urban areas. Region-wise, the highest infant deaths were found in the central region (6.79), while a significant preponderance of child deaths was found in the central region of India (2.05).

**Table 1:** Percentage of Infant and child deaths by their background characteristics in India, 2015–16 (*n* = 824,693)

***Bio-Demographic Factors***	Infant deaths (0–11 months)	Child deaths (12–59 months)
Survival status of any preceding child’s		
**Alive**	3.83	1.20
**Dead**	15.21	2.86
Sex of the child		
**Male**	4.98	1.12
**Female**	4.73	1.61
Maternal age at childbirth		
**< 18**	5.61	1.63
**18–34**	4.57	1.24
**35+**	4.68	0.91
Child’s Birth order		
**2**	4.09	1.03
**3**	4.79	1.38
**4+**	6.43	1.93
Preceding birth interval		
**< 19 months**	10.22	2.36
**19–23 months**	5.49	1.68
**24–35 months**	4.07	1.34
**36+ months**	2.47	0.72
Number of births		
**Single**	4.45	1.34
**Multiple**	26.61	1.82
***Maternal socioeconomic factors***		
Education		
**Illiterate**	5.94	1.92
**Primary**	4.76	1.16
**Secondary**	3.36	0.56
**Higher**	2.08	0.23
Religion		
**Hindu**	5.04	1.39
**Muslim**	4.44	1.23
**Others**	3.49	1.05
Caste		
**Deprived**	5.31	1.67
**Not-Deprived**	4.74	1.26
Wealth Index		
**Poorest**	6.44	2.21
**Poorer**	5.40	1.54
**Middle**	4.61	1.04
**Richer**	3.75	0.85
**Richest**	2.86	0.52
Type of Residence		
**Urban**	3.76	0.93
**Rural**	5.29	1.51
Regions		
**North**	4.26	1.21
**Central**	6.79	2.05
**East**	4.85	1.40
**North-East**	4.35	1.24
**West**	3.13	0.73
**South**	3.45	0.67
**Total**	**4.86**	**1.35**

Table 2 predicts the results of the hazard models on the determinants of infant mortality**.** In model 1, the partial effect of survival status of the preceding child was strong as the infants with the preceding child as dead were more likely to experience infant mortality (3.43*) than the alive ones. Model 2 includes bio-demographic factors along with the survival status of the preceding child. After the inclusion of other factors, the effect of survival status on infant death reduces to a certain extent (2.63*). Female children were more likely to experience infant mortality than their male counterparts (1.16*). Higher maternal age at childbirth (18–34) depicted a lower likelihood of infant deaths (0.85*) than the below 18 age group. Children born after a birth interval of 36+ months were less likely to experience infant mortality (0.61*) compared to a birth interval of less than 19 months. Lastly, multiple births showed more likelihood of infant mortality (4.56*) than single births. Model 3 includes the survival status of the preceding child along with the maternal socioeconomic factors. In model 3, the effect of the survival status of the preceding child on infant mortality remains substantial (3.08*), implying that infant mortality was more likely to occur among the births with a preceding dead child. Among these factors, place of residence came up as insignificant while the education of mother showed a negative relationship with infant mortality. It was seen that infants of educated mothers experienced lower mortality (0.46*) compared to infants whose mothers were illiterate. Infants belonging to other religions were more likely to experience mortality (1.13*) than their Hindu counterparts. Infant deaths were less likely to occur among the not-deprived caste category (0.96*) than the deprived ones. The household wealth index showed a negative association with infant mortality wherein the children from the wealth quintile were least likely to experience infant mortality than the poorest ones. Lastly, infants from the central region of India were more likely to experience mortality (1.34*), whereas the least likelihood was found in the western region (0.67*) compared to the northern region.

**Table 2:** Results of Cox proportional hazards models (hazard ratio) on the determinants of infant mortality (0–11 months) in India, NFHS 2015–16

***Bio-Demographic Factors***	Model 1	Model 2	Model 3	Model 4
Survival status of any preceding child’s	**Hazard Ratio**	**Hazard Ratio**	**Hazard Ratio**	**Hazard Ratio**
**Alive®**	Ref.	Ref.	Ref.	Ref.
**Dead**	3.43*(3.3,3.57)	2.63*(2.52,2.74)	3.08*(2.96,3.21)	2.42*(2.32,2.53)
Sex of the child				
**Male®**		Ref.		Ref.
**Female**		1.16*(1.12,1.20)		1.15*(1.11,1.19)
Maternal age at childbirth				
**< 18®**		Ref.		Ref.
**18–34**		0.85*(0.82,0.88)		0.86*(0.83,0.89)
**35+**		0.80*(0.67,0.97)		0.83(0.69,1.00)
Child’s Birth order				
**2®**		Ref.		Ref.
**3**		1.20*(1.15,1.25)		1.11*(1.06,1.16)
**4+**		1.53*(1.47,1.59)		1.29*(1.24,1.34)
Child’s Birth interval				
**< 19 months**		Ref.		Ref.
**19–23 months**		0.61*(0.58,0.65)		0.60*(0.57,0.64)
**24–35 months**		0.48*(0.46,0.5)		0.47*(0.45,0.5)
**36+ months**		0.29*(0.27,0.3)		0.29*(0.27,0.3)
Multiple births				
**Single®**		Ref.		Ref.
**Multiple**		4.56*(4.22,4.94)		4.77*(4.41,5.17)
***Maternal socio-economic factors***				
Education				
**Illiterate®**			Ref.	Ref.
**Primary**			0.88*(0.84,0.92)	0.89*(0.85,0.93)
**Secondary**			0.71*(0.68,0.75)	0.75*(0.72,0.79)
**Higher**			0.46*(0.39,0.54)	0.54*(0.46,0.63)
Religion				
**Hindu®**			Ref.	Ref.
**Muslim**			0.95(0.91,1)	0.89*(0.85,0.94)
**Others**			1.13*(1.06,1.21)	1.09*(1.01,1.17)
Caste				
**Deprived®**			Ref.	Ref.
**Not-Deprived**			0.96*(0.92,1)	0.98(0.94,1.02)
Wealth Index				
**Poorest®**			Ref.	Ref.
**Poorer**			0.93*(0.89,0.97)	0.93*(0.88,0.97)
**Middle**			0.89*(0.85,0.94)	0.88*(0.84,0.93)
**Richer**			0.83*(0.77,0.88)	0.82*(0.77,0.88)
**Richest**			0.68*(0.62,0.74)	0.70*(0.65,0.76)
Type of Residence				
**Urban®**			Ref.	Ref.
**Rural**			1.00(0.95,1.05)	1.01(0.96,1.06)
Regions				
**North®**			Ref.	Ref.
**Central**			1.34*(1.27,1.41)	1.33*(1.26,1.40)
**East**			0.9*(0.85,0.96)	0.93*(0.87,0.99)
**North-East**			1.02(0.95,1.10)	1.09*(1.01,1.17)
**West**			0.67*(0.61,0.74)	0.67*(0.61,0.74)
**South**			0.72*(0.66,0.78)	0.73*(0.67,0.8)

**®/Ref:** Reference group; *if *p* < 0.05.

Model 4 includes all the factors into consideration, i.e., bio demographic and maternal socioeconomic factors. With the inclusion of all the factors, the effect of survival status of the preceding child decreases but remains significant (2.42*). Factors like caste and place of residence became insignificant in this full effect model. Like model 2, female infants were more likely to experience mortality (1.15*) as compared to male infants. Infants born to mothers of age 18–34 showed less likelihood of mortality (0.86*) as compared to mothers aged less than 18 years. Similar to model 2, infants with a birth interval of more than 36 months were less likely to die (0.29*) when compared to infants with a birth interval of less than 19 months. Education of mother and household wealth index showed a negative relationship with infant deaths.

Infants from the central region of India were more likely to experience mortality (1.33*) as compared to infants from the northern region. In contrast, the least likelihood of infant mortality was found in the western region (0.67*) compared to the northern region.

The results of the hazard models on the determinants of child mortality are shown in Table 3**.** In model 1, the risk of dying in early childhood is about 2.79 times higher among children with a preceding dead child. This result changes marginally in model 2, where the risk of dying in early childhood is about 2.27 times higher among children with a preceding dead child. There is a significant difference between male and female child mortality; the latter is associated with statistically significant excess early childhood mortality (1.37*) compared to male children. Children born to mothers of age 18–34 years had a mortality rate that is nearly 20% lower (0.80*) than their counterparts. Children born at 4+ birth order experienced higher mortality than their respective counterparts (1.61*). Children born with a birth interval of more than 36+ months were 66% less likely to experience child mortality (0.34*). Multiple births continue to experience a mortality rate that is 1.72 times higher than single births. In model 3, the effect of the survival status of the preceding child was significantly associated with child mortality (2.33*). Mother’s education was significantly related to the risk of death among children as children whose mothers had higher education status were 63% less likely to experience child mortality than their counterparts. Children from other religions showed a 19% more likelihood of experiencing mortality than their Hindu counterparts. Lower risk of child mortality was observed among children belonging to the non-deprived section of the society (0.89*). Household wealth was negatively associated with child deaths as the richest children were 58% less likely to experience child mortality than the poorest lot. Region-wise association showed that children belonging to the central region were 56% more likely to experience child mortality than the children in the northern region. Model 4 is the full effect model, including all the factors, shows that the effect of survival status of preceding child on child mortality has decreased but is still significant (1.95*). The pattern of difference between male and female child mortality remains the same as model 2, where female gender was associated with statistically significant excess early childhood mortality (1.36*). Mother’s age at birth emerged to be significantly related to the risk of death in childhood, thereby depicting a lower risk of child mortality (0.68*) when the mother’s age was above 35 years. In the same model 2, children born at 4 + birth order experienced higher mortality than their respective counterparts (1.19*). Children born with a birth interval of more than 36+ months were 66% less likely to experience child mortality (0.34*).

**Table 3:** Results of Cox proportional hazards models (hazard ratio) on the determinants of child mortality (12–59 months) in India, NFHS 2015–16

***Bio-Demographic Factors***	Model 1	Model 2	Model 3	Model 4
Survival status of any preceding child’s	**Hazard Ratio**	**Hazard Ratio**	**Hazard Ratio**	**Hazard Ratio**
**Alive®**				
**Dead**	2.79*(2.67,2.92)	2.27*(2.16,2.37)	2.33*(2.23,2.44)	1.95*(1.86,2.04)
Sex of the child				
**Male®**				
**Female**		1.37*(1.33,1.43)		1.36*(1.31,1.41)
Maternal age at childbirth				
**< 18®**				
**18–34**		0.80*(0.77,0.83)		0.83*(0.80,0.86)
**35+**		0.65*(0.52,0.81)		0.68*(0.54,0.85)
Child’s Birth order				
**2®**				
**3**		1.26*(1.20,1.32)		1.1*(1.05,1.15)
**4+**		1.61*(1.54,1.68)		1.19*(1.14,1.24)
Child’s Birth interval				
**< 19 months**				
**19–23 months**		0.78*(0.74,0.82)		0.75*(0.71,0.79)
**24–35 months**		0.60*(0.58,0.63)		0.58*(0.56,0.61)
**36+ months**		0.34*(0.32,0.36)		0.34*(0.32,0.36)
Multiple births				
**Single®**				
**Multiple**		1.72*(1.50,1.96)		1.85*(1.62,2.12)
***Maternal socioeconomic factors***				
Education				
**Illiterate®**				
**Primary**			0.72*(0.68,0.76)	0.73*(0.69,0.77)
**Secondary**			0.50*(0.47,0.53)	0.53*(0.50,0.56)
**Higher**			0.27*(0.22,0.35)	0.33*(0.26,0.42)
Religion				
**Hindu®**				
**Muslim**			0.93*(0.88,0.98)	0.88*(0.83,0.93)
**Others**			1.19*(1.10,1.28)	1.15*(1.06,1.24)
Caste				
**Deprived®**				
**Not-Deprived**			0.89*(0.86,0.93)	0.91*(0.87,0.95)
Wealth Index				
**Poorest®**				
**Poorer**			0.79*(0.76,0.83)	0.79*(0.75,0.82)
**Middle**			0.64*(0.6,0.68)	0.63*(0.60,0.67)
**Richer**			0.54*(0.51,0.59)	0.54*(0.51,0.59)
**Richest**			0.42*(0.38,0.47)	0.43*(0.39,0.48)
Type of Residence				
**Urban®**				
**Rural**			0.96(0.91,1.02)	0.97(0.92,1.03)
Regions				
**North®**				
**Central**			1.56*(1.48,1.66)	1.55*(1.46,1.65)
**East**			1.05(0.98,1.12)	1.08*(1.01,1.15)
**North-East**			1.08(0.99,1.17)	1.13*(1.04,1.23)
**West**			0.81*(0.73,0.90)	0.80*(0.72,0.89)
**South**			0.71*(0.65,0.79)	0.72*(0.65,0.80)

**®/Ref:** Reference group; *if *p* < 0.05.

Multiple births continue to experience a mortality rate that was 1.85 times higher than single births. Household wealth and education of mothers proved to be strong predictors of child mortality. Children from the richest households and born to educated mothers had a child mortality rate of about 57% and 77% lower than their counterparts from the poorest economic class and illiterate mothers, respectively. Region emerged as a strong predictor of infant mortality, with births from the central region recording child mortality about 55% higher than those from the north region.

**Fig. 1:**
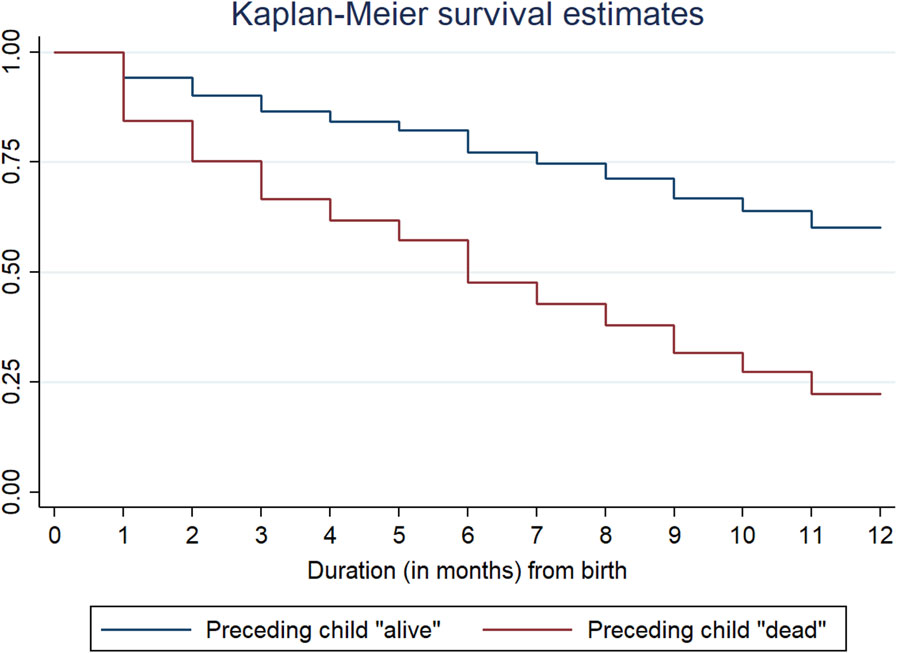
Kaplan-Meier survival estimates depicting survival curve for Infant mortality (0–11 months) by survival status of any preceding child, 2015–16**.** Duration in months (from birth)

**Fig. 2:**
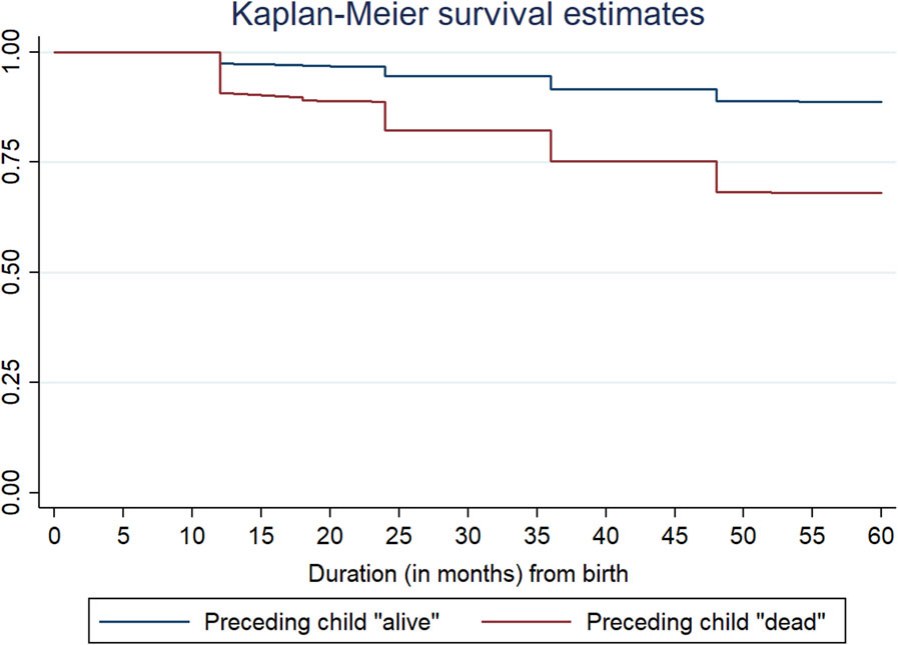
Kaplan-Meier survival estimates depicting survival curve for child mortality by survival status of any preceding child, 2015–16**.** Duration in months (from birth)

Infant and child mortality trajectories by preceding child survival status were depicted in Fig. 1 and Fig. 2, respectively. It was found that the probability of infant and child mortality was high among those children whose previous sibling was dead.

## Discussion

This study shows how the preceding child’s survival status affects the survival status of the succeeding child. Other maternal bio-demographic and demographic factors are taken into consideration to carry out the analysis.

The socio-economic status of the mother plays a vital role in determining the survival status of the child. As found in the present study and evidence from other literature, infant and child survival is low among illiterate and poor mothers [35]. In the present study, it was found that the survival status of the preceding child plays a vital role in the survival status of the succeeding child. Similar results were depicted in other studies, too [36, 37]. Mosley & Chen’s analytical framework for studying the determinants of child survival also found age, parity, and birth interval as determining factors for child survival [38]. It has been found that the effect of short preceding birth intervals on child mortality is strong when a preceding sibling dies [39]. This statement signifies that if the preceding birth interval is short, then chances of infant or child death are high, and if the child dies, then this forms a chain of death clustering among siblings having short PBI and dead siblings.

Over the last few years, hyped attention has been given to children born to mothers having a higher risk of repeated child loss. Researchers started taking an interest in related studies during the late 1930s when in 1936, Gardiner and Yerushalmy identified a repetitive pattern of fetal losses occurring to the same set of women [40]. Thereafter, another study following the suit examined that in mothers whose last pregnancy terminated in fetal or infant death, there was a tendency for the death to recur [41–44]. Studies carried out in developing countries also highlighted the association between previous child mortality and survival of subsequent children; and noted a lower risk of neonatal and postnatal mortality if the previous child survived to at least one year of age than if it was dead during infancy [45–47]. In the Indian context also, previous research highlighted a higher proportion of children dying to the mothers whose previous child was dead [48].

The results noted an increased risk of infant and child mortality among those whose previous sibling was dead. Several factors such as a lower level of mother’s education predispose the children to higher death risk during subsequent birth history [49]. Why would the death of a child leads to a higher risk of death for the next born child of the same mother is a very critical query to seek an answer. One plausible mechanism operates by the death of a child, shortening the time to the next birth. One possibility, also known as the fecundity hypothesis, is that the death of an infant results in the mother ceasing to breastfeed, and so they can conceive sooner than otherwise [50, 51]. The alternate hypothesis, known as the replacement hypothesis, states that the death of a child leads parents to intentionally conceive sooner in a desire to replace their loss [52]. Following both the hypothesis mentioned above, it is clear that a shorter birth interval for the index child followed by the death of the previous child is a risk factor that could cause an elevated risk of death [53]. Several previous studies have outlined the risk of child death associated with shorter birth intervals [53, 54]. However, a study noted that a child born after a short birth interval has higher chances to survive its infancy if the previous sibling died than if it survived [55]. A shorter birth interval history is more damaging to poor women, and the risk of child death is even higher [56]. This is so because poor women might be undernourished, and it takes time to recover physiologically from birth before the women could be ready for the subsequent pregnancy. Moreover, child death leaves a mother depressed, leading to compromise in the subsequent birth if the birth interval is shorter, leading to an increased risk of child death, known as the depression hypothesis [49].

First, women with short birth intervals have shorter recuperative intervals between the end of lactation and the start of a new pregnancy than women with longer intervals. The over-lap of gestation and lactation is particularly stressful to the mother and the child. Therefore, the joint effects of pregnancy and lactation on a mother’s physiology and nutritional status may be a mechanism through which short preceding intervals affect child-health status. This may result in low birth- weight children, prematurity, or impaired lactational abilities. Second, short birth intervals are associated with a higher risk of not attending prenatal care at all. This higher risk may be due to the lack of opportunities to attend antenatal clinics since there is a young child to care for, although controlling for the survival status of the previous child did not affect it. There may also be sociocultural or personal reasons for not attending prenatal care since a rapid return to pregnancy is often considered undesirable and perhaps embarrassing. Another explanation is that a higher proportion of premature deliveries among women having short birth intervals contributes to the lower proportion attending prenatal care among such women, particularly if women tend to make the first visit to antenatal clinics late in pregnancy. Women with long birth intervals (of three years or more) have moderately higher levels of attendance at prenatal care and are slightly more likely to deliver in institutions than women with shorter intervals. Both more opportunities and better motivation to have another child may contribute to these differences [12]. Children born with multiple births were more prone to die in infancy than those born as single children. Other studies also confirm that multiple births such as twins or triplets are at high risk of pregnancy and childbirth [57].

Similar to earlier studies, it was found that central India exhibits the highest IMR and child mortality, whereas southern and western regions perform better in the respective scenario [5]. It was further commented that IMR was higher in north India in comparison to south India. The bivariate and multivariate analysis show that illiteracy, working status of women, and low age at birth were the main mother-related covariates for a high IMR [58]. Girls die more commonly than boys due to existing underlying causes like inequality in health care; for example, fewer girls are vaccinated in health facilities [59]. RMNCH+A interventions were rolled out to achieve under-five mortality to 33/1000 and IMR to 25/1000 by 2017; however, the goals have not been achieved yet. The current rate of under-five and infant death is 39 and 34 per 1000, respectively, far behind the goals to be achieved [60]. Younger maternal age is associated with lower birth weight, preterm birth, and low nutritional status, leading to a higher risk of infant and child mortality. The children of teenage mothers aged ≤19 years had higher chances of preventable child death. Women belonging to low socio-economic tend to get pregnant early and find it challenging to take care of newborns due to poverty [61]. Finding from other studies also adds that the risk of under-five death is high among women aged 16 years or less [62, 63].

Infant and child deaths are highly clustered among those mothers whose earlier child was dead. This signifies that some mothers are always at a disadvantageous stage in infant and child mortality. Moreover, as maternal childbearing age is still low in India, it poses a high risk of infant and child death. Mothers with higher birth order have a high risk of losing their children as it is challenging to take care of every child.

One of the study’s main limitations is the spacing between live births, i.e., the definition of interval ignores the abortion ((both induced and spontaneous) and stillbirths. It leads to difficulty as both reproductive failures and intentional pregnancy spacing are associated with increased intervals between live births. Another major limitation could be the unavailability of the sex history of the previous child who was dead. If sex were to be known, the study would have sought the attention of policymakers, given the high sex disparity in the country.

## Conclusion

Education is a way out, and there is a need to focus on girl’s education. The government shall also focus on raising awareness on the importance of spacing between two successive births, as this will undoubtedly lower infant mortality and child mortality. There is also a need to create a better health infrastructure catering to the needs of rich and poor people alike. There is also a need to strengthen the current health system in India for safe perinatal care with community engagement. It has been noted in a previous study that the current numbers of sub-centres, PHCs, and CHCs are not sufficient to meet their population norms, and therefore there is a need to increase the number of such facilities across the country [24]. There is also a need for such interventions that would cater to maternal healthcare needs to improve child survival by upgrading infrastructure and improving human resource support during delivery care.

## Data Availability

The study utilizes secondary sources of data freely available in the public domain https://dhsprogram.com/methodology/survey/survey-display-355.cfm. The necessary ethical approval has been taken by the respective organisations involved in the data collection process.

## Abbreviations

MDG: Millennium Development Goals
WHO: World Health Organization
U5MR: Under-five Mortality Rate
IMR: Infant Mortality Rate
UNICEF: United Nations International Children’s Emergency Fund
NFHS: National Family Health Survey
MoHFW: Ministry of Health and Family Welfare
IIPS: International Institute for Population Sciences
PBI: Preceding Birth Interval
SC: Scheduled Caste
ST: Scheduled Tribe
RMNCH + A: Reproductive, Maternal, Newborn, Child and Adolescent Health
PHC: Primary Health Centre
CHC: Community Health Centre
